# The complete mitochondrial genome sequence of *Gerres filamentosus* (Perciformes: Gerridae)

**DOI:** 10.1080/23802359.2017.1413298

**Published:** 2018-02-01

**Authors:** Wei Shi, Baosheng Wu, Hui Yu

**Affiliations:** a College of Life Science, Foshan University, Foshan, Guangdong, China;; b CAS Key Laboratory of Tropical Marine Bio-resources and Ecology, South China Sea Institute of Oceanology, Chinese Academy of Sciences, Guangzhou, China

**Keywords:** Complete mitochondrial genome, *Gerres filamentosus*, phylogenetic relationship

## Abstract

The complete mitochondrial genome of *Gerres filamentosus* was sequenced by high throughput sequencing method. Length of this genome is 16,795 bp, containing 13 protein-coding genes, 22 tRNA genes, two rRNA genes and one large non-coding region. *ND6* and eight tRNA genes are encoded by L-strand, and others are encoded by H-strand, which is similar to those in most vertebrates. Phylogenetic tree based on 13 protein-coding genes shows that the clade of *G. filamentosus* is closely clustered with that of *Gerres oyena*, and families Caproidae and Ephippidae have the closest relationship to Gerridae, comparing with Sillaginidae

Gerridae fishes are marine species (occasionally brackish and rarely in freshwater) inhabiting in warm seas. *Gerres filamentosus* is widely distributed in tropical and sub-tropical waters of worldwide. Gerridae species has been confused in taxonomic status for long time, because of their similar morphological characters and coloration (Heemstra and Yukio 2007). In this paper, we got the sample from Naozhou island in Zhanjiang (geographic coordinate: N 20°53′20.11″, E 112°28′46.2″). The whole body specimen was preserved in ethanol and registered to the Marine Biodiversity Collection of South China Sea, Chinese Academy of Sciences, under the voucher number SCF20171022002. It is the first reported about the complete mitogenome of *G. filamentosus*, and analyzed its phylogenetic relationships within order Perciformes.

The complete mitochondrial genome of *G. filamentosus* has 16,795 bp in length (GenBank accession no. MG386479), including 13 protein-coding genes, two ribosomal RNA (rRNA) genes, 22 transfer RNA (tRNA) genes and one control region (D-Loop). Genes encoding on the genome are similar to other vertebrates (Yu and Kwak 2015). The OL (origin of replication on the light-strand) was found in the cluster of five tRNA genes (*WANCY* region) between *tRNA-Asn* and *tRNA-Cys*. And the D-Loop (control region) is 956 bp in length, between *tRNA-Pro* and *tRNA-Phe*. Among 13 protein-coding genes, 12 ORF started with ATG and rest one (COI) started with GTG, which is as same as *Liobagrus styani* (Huang et al. 2017). However, for the stop codon, *ND4* and *Cytb* ended with just a single base T, which is different from the common stop codon with three bases. But the same situation was also found in many other fishes, such as *Liobagrus styani* (Huang et al. 2017), *Brachymystax lenok tsinlingensis* (Yu and Kwak 2015). Among above genes, except *ND6* and eight tRNA genes were encoded on L-strand, others were encoded on H-strand, which is similar to those of other vertebrates. The base composition of genome of *G. filamentosus* is T: 25.9%, C: 30.0%, A: 27.6% and G: 16.5%.

The phylogenetic relationships of *G. filamentosus* with 10 closely related species were analyzed in this study. The complete mitochondrial genes of these ten species are available on GenBank. The maximum-likelihood evolutionary tree (ML tree) was constructed by MEGA 7 (Kumar et al. 2016) based on 1st and 2nd codon sequences of 13 protein-coding genes. In the ML tree, *G. filamentosus* clustered with *G. oyena* with a strong support, *A. capro, P. orbicularis and P. teira* formed a clade as a sister lineage to clade of *G. filamentosus* and *G. oyena* (Figure 1). These results show that Caproidae and Ephippidae have closer phylogenetic relationship to Gerridae than to Sillaginidae.

**Figure 1.**
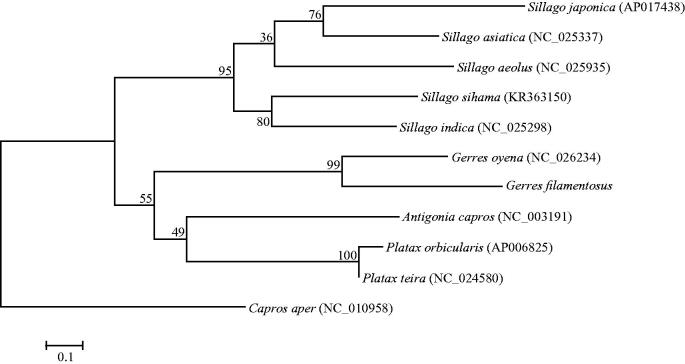
Maximum-likelihood tree was constructed based on 1st and 2nd codon sequences of 13 protein-coding genes of 11 species.
